# Supplementary material for: MetaRibo-Seq measures translation in microbiomes
Source: Nat Commun. 2020 Jun 29;11:3268. doi: 10.1038/s41467-020-17081-z (PMC7324362; doi:10.1038/s41467-020-17081-z)
Supplement: Supplementary file 10 — Supplementary Data 7 [file 41467_2020_17081_MOESM10_ESM.zip › File2/Confidence_High_Taxonomy/367970_out.krona.html]

Javascript must be enabled to view this page.

members
magnitude
magnitudeUnassigned
count
unassigned
taxon
rank

367970\_out

112

112
superkingdom
2

112
phylum
976

112
class
200643

order
112
171549

815
family
112

112
genus
816

species
1

SRS075984\_contig\_number\_12216
246787

species
1

SRS077335\_contig\_number\_1419
817

108
species
47678

SRS012273\_contig\_number\_48420SRS012849\_contig\_number\_28382SRS013476\_contig\_number\_39574SRS013521\_contig\_number\_10554SRS013951\_contig\_number\_contig-100\_919.231913SRS014287\_contig\_number\_11402SRS014412\_contig\_number\_21204SRS014613\_contig\_number\_6334SRS014683\_contig\_number\_6947SRS014923\_contig\_number\_26568SRS015264\_contig\_number\_10890SRS015663\_contig\_number\_31505SRS015782\_contig\_number\_40932SRS016056\_contig\_number\_1008SRS016989\_contig\_number\_16126SRS017433\_contig\_number\_9742SRS017701\_contig\_number\_398SRS017821\_contig\_number\_18191SRS018049\_contig\_number\_40SRS018427\_contig\_number\_14280SRS018623\_contig\_number\_2892SRS018836\_contig\_number\_14538SRS018888\_contig\_number\_7455SRS019030\_contig\_number\_4173SRS019161\_contig\_number\_14470SRS019267\_contig\_number\_6192SRS019910\_contig\_number\_6921SRS020328\_contig\_number\_31883SRS022137\_contig\_number\_11505SRS024132\_contig\_number\_24199SRS024492\_contig\_number\_12175SRS042966\_contig\_number\_15737SRS043001\_contig\_number\_77SRS043667\_contig\_number\_4264SRS043768\_contig\_number\_14775SRS043841\_contig\_number\_4340SRS044535\_contig\_number\_19981SRS045244\_contig\_number\_447SRS045244\_contig\_number\_768SRS045826\_contig\_number\_27SRS046068\_contig\_number\_4801SRS047044\_contig\_number\_12446SRS048164\_contig\_number\_29802SRS049773\_contig\_number\_4375SRS049995\_contig\_number\_29594SRS050299\_contig\_number\_12758SRS050752\_contig\_number\_24895SRS050925\_contig\_number\_17151SRS050998\_contig\_number\_15339SRS051882\_contig\_number\_19359SRS052078\_contig\_number\_211SRS053649\_contig\_number\_16532SRS054905\_contig\_number\_10018SRS058723\_contig\_number\_2583SRS058770\_contig\_number\_9017SRS062427\_contig\_number\_7495SRS064276\_contig\_number\_16370SRS064557\_contig\_number\_11203SRS064757\_contig\_number\_24932SRS065176\_contig\_number\_2280SRS075878\_contig\_number\_12375SRS077024\_contig\_number\_9858SRS077086\_contig\_number\_12757SRS077454\_contig\_number\_6080SRS077589\_contig\_number\_contig-100\_1536.50821SRS078176\_contig\_number\_22965SRS078242\_contig\_number\_27847SRS078419\_contig\_number\_36726SRS078665\_contig\_number\_422SRS1041039\_contig\_number\_24416SRS1041090\_contig\_number\_1728SRS1041091\_contig\_number\_9376SRS1041092\_contig\_number\_16330SRS1041118\_contig\_number\_14333SRS1041137\_contig\_number\_30876SRS1041138\_contig\_number\_14437SRS1041141\_contig\_number\_14388SRS1041157\_contig\_number\_3044SRS104311\_contig\_number\_21082SRS104327\_contig\_number\_4286SRS1054716\_contig\_number\_16223SRS1055049\_contig\_number\_11582SRS142923\_contig\_number\_3675SRS143181\_contig\_number\_14055SRS143342\_contig\_number\_11175SRS143523\_contig\_number\_379SRS143598\_contig\_number\_136SRS143722\_contig\_number\_952SRS143895\_contig\_number\_41905SRS144362\_contig\_number\_21005SRS145497\_contig\_number\_553SRS147557\_contig\_number\_32427SRS147614\_contig\_number\_9586SRS147977\_contig\_number\_4504SRS148091\_contig\_number\_13542SRS148196\_contig\_number\_contig-100\_1149.256419SRS148253\_contig\_number\_9511SRS148817\_contig\_number\_4161SRS149181\_contig\_number\_15028SRS149244\_contig\_number\_6737SRS149784\_contig\_number\_18644SRS149879\_contig\_number\_23680SRS893279\_contig\_number\_contig-100\_1425.99452SRS893285\_contig\_number\_1275SRS893327\_contig\_number\_7875SRS893378\_contig\_number\_6264SRS893382\_contig\_number\_84SRS893383\_contig\_number\_23698

1
species

SRS022071\_contig\_number\_36589
28116

818

SRS142542\_contig\_number\_5891
species
1
